# Supplementary material for: Brain Tumor-Induced Changes in Routine Parameters of the Lipid Spectrum of Blood Plasma and Its Short-Chain Fatty Acids
Source: Curr Issues Mol Biol. 2025 Mar 26;47(4):228. doi: 10.3390/cimb47040228 (PMC12026183; doi:10.3390/cimb47040228)
Supplement: Supplementary file 1 [file cimb-47-00228-s001.zip › Supplementary information File S1.pdf]

Supplementary information

Additional File S1 of BRAIN TUMOR-INDUCED CHANGES IN ROUTINE PARAMETERS OF THE LIPID SPECTRUM OF BLOOD PLASMA AND SHORT-CHAIN FATTY ACIDS.

Supplementary Table S1. Clinicopathological features of patients

	Age		Gender		Mean tumor volume (cm^3)	Mean BMI	% of smokers in the group
	< 60	≥ 60	Male	Female			
"Healthy" group (control)	38	12	16	34	-	69	24
Atherosclerosis (n=50)	9	41	38	12	-	27.48	87
Brain tumors, Grade I (n=1)	1	-	-	1	5.21	76	-
Brain tumors, Grade II (n=6)	5	1	1	5	27.5	80	20
Brain tumors, Grade III (n=11)	8	3	4	7	57.8	66.6	25
Brain tumors, Grade IV (n=32)	11	21	15	17	44.3	75	12.5
