# Supplementary material for: Brain Tumor-Induced Changes in Routine Parameters of the Lipid Spectrum of Blood Plasma and Its Short-Chain Fatty Acids
Source: Curr Issues Mol Biol. 2025 Mar 26;47(4):228. doi: 10.3390/cimb47040228 (PMC12026183; doi:10.3390/cimb47040228)
Supplement: Supplementary file 1 [file cimb-47-00228-s001.zip › Supplementary information File S2.pdf]

Supplementary information
Additional File S2 of BRAIN TUMOR-INDUCED CHANGES IN ROUTINE PARAMETERS OF THE LIPID SPECTRUM OF BLOOD PLASMA AND SHORT-CHAIN FATTY ACIDS

Local Ethics Committee
Federal State Budgetary Educational Institution of Higher Education Pedagogical Medical University of the Ministry of Health of the Russian Federation
Address: 603005 Nizhny Novgorod, Minin and Pozharsky Square, 10/1

Informed consent form

The research is carried out by Doctor	Medyanik Igor Alexandrovich,<br>+7-908-767-62-21
Purpose of the study	A. Analyze the metabolic features in pathologies caused by activation of cell proliferation;<br>B. Develop methods for screening diagnosis of neoplasms based on the obtained biochemical parameters of blood plasma.
New diagnostic method	A new screening diagnostic method, the effectiveness of which is tested in comparison with existing examination methods.
Other possible methods of diagnosing your disease:	List the main diagnostic methods available at the moment: the content of tumor markers in the blood, computed tomography, ultrasound.
To participate in this study, in addition to your usual course of treatment (examination), you must:	Go through routine tests like other patients, but during the collection an additional volume of blood of 10 ml will be taken. The areas of the brain, peritumoral zone and part of the tumor that are removed during surgery and usually disposed of will be subjected to the above study.
Test period:	During preoperative preparation in patients with initially diagnosed brain tumors.
Possible risks:	There are no health risks. If you refuse to participate in the study, you will be treated as usual according to the approved plan without restrictions.
In case of emergency, please contact:	Medyanik Igor Alexandrovich,<br>+7-908-767-62-21<br>Obukhova Larisa Mikhailovna,<br>+7-951-914-55-45
Expected effect of treatment:	Developing a way to screen patients with suspected cancer that may be more effective than previously used screening methods and that may benefit you during or after the trial, and that your participation in the trial may help other patients.
Your health information is confidential and can only be viewed by authorized persons.	
You have every right:	Refuse to participate in this study, or, if you agree, change your mind at any time, and your withdrawal will not have an impact on your future medical care.

Date

Patient's signature _______________/

PATIENT INFORMATION

Study title: studying metabolic parameters in patients with brain tumors and developing a method for early diagnosis of brain tumors.

1. **Introduction**

Local Ethics Committee
Federal State Budgetary Educational Institution of Higher Education Pedagogical Medical University of the Ministry of Health of the Russian Federation
Address: 603005 Nizhny Novgorod, Minin and Pozharsky Square, 10/1

You are invited to take part in a scientific study that is being conducted at the Federal State Budgetary Institution "NNIITO" of the Ministry of Health and Social Development of Russia

The information in the “PATIENT INFORMATION” is very important because it tells you what the study is about. Please read it carefully and make sure you fully understand the information it contains. If you have any questions, your doctor will try to answer them. After you have read this document and expressed your desire to participate in this study, you will be asked to sign the “INFORMED CONSENT FORM”. The “INFORMED CONSENT FORM” you sign will confirm that you have received full information about the study and agree to participate in it.

One copy of the PATIENT INFORMATION, dated and signed by the physician investigator, will be given to you. Please do not rush into making a decision about participating in this study. You can take as much time as you need to think about whether or not you want to participate in this study.

2. **What is the purpose of this study?**

A. To analyze the metabolic features in pathologies caused by activation of cell proliferation;
B. To develop methods for screening diagnostics of neoplasms based on the obtained biochemical parameters of blood plasma.

The aim of this study is to determine the relationship between metabolic changes in the tumor, peritumoral zone and in unchanged brain tissue.

The information obtained in this study will be processed and analyzed to determine changes in enzyme activity depending on the degree of tumor malignancy. Your personal data will be kept confidential (anonymized). This information may be used in reports on the study or in scientific papers. Your name will not be mentioned and your anonymity will be maintained in all reports or scientific papers.

Patient's signature _________/   /   Date _________________

1. **How many people will take part in this study??**

This study, conducted at the Federal State Budgetary Educational Institution of Higher Education “PIMU” of the Ministry of Health of Russia, will involve 20 people.

2. **Should I participate?**

It is up to you to decide whether to take part in this study or not. You should not feel obliged to do so. If you wish. You can think it over quietly at home, or consult with your doctor or relatives. You can refuse to take part in the study at any time, without giving a reason. This will not affect your further treatment in any way.

3. **What will happen to me if I agree to participate in the study?**

During brain tumor surgery, in order to provide adequate access to the tumor, it is often necessary to remove some small functionally insignificant areas of the brain, which are then disposed of. In addition, it is known that malignant brain tumors grow by infiltrating the surrounding brain tissue. After the

tumor is removed, in order to remove the remaining tumor cells in this area surrounding the tumor (peritumoral), these areas of the brain are also removed within the limits that do not cause motor or speech or any other functions, all this is done to preserve your quality of life.

The areas of the brain, peritumoral zone and part of the tumor that are removed during the operation and usually disposed of will be subjected to the above-mentioned examination..

Duration of the study

The total duration of the study is the time the patient stays in the neurosurgical hospital.

Research procedures

If you express a desire to participate in this study and sign the Informed Consent, your doctor will conduct several standard examinations. These examinations will be conducted to make sure that you are suitable for participation in this study and that your participation in the study is safe for you. Before the operation, your doctor will ask you questions about your general health, past illnesses, medications you are taking, and bad habits. A standard medical examination will be performed, which includes a CT scan, functional MRI and tractography if the tumors are located in functionally significant areas of the brain, general clinical, biochemical tests, and blood clotting..

If it is decided that you are not suitable for participation in this study, you will receive standard treatment.

4. **What should I do?**

Please tell your doctor what medications you are taking. This is for safety and comfort, as some medications may interfere with each other.

Make sure you tell us about all concomitant diseases and previous (surgical) operations. Participation in the study does not require changing the nature of nutrition and lifestyle during the study period.

5. **What methodology is being studied?**

It is known that during tumor growth, certain changes in metabolic processes occur in the tumor itself and the peritumoral zone. These changes, in turn, can affect the further development of the disease. The study examines the relationship between metabolic changes and the degree of tumor anaplasia and molecular genetic changes, which are prognostic indicators.

6. **What side effects may occur during treatment? What risks do I have by participating in the study?**

Risks are normal for standard brain tumor removal. There are no additional risks if you participate in the study. Since only those removed tissues that are usually disposed of after tumor removal will be examined. And in this case, they will be studied.

7. **What are the benefits of participating in the study?**

The information obtained in this study will help us to improve our understanding of the development of brain tumors. This may eventually have an impact on improving the methods of treatment of this pathology.

8. **What if something happens?**

We do not expect any problems with your health related to the use of the method under study. However, in case of proven deterioration of your health, you will receive qualified medical care free of charge at the Federal State Budgetary Educational Institution of Higher Education "PIMU" of the Ministry of Health of Russia.

It is important that you inform your doctor as soon as possible about any changes in your health.

Patient's signature ______ Date _________________

9. Will my participation in the study be kept confidential?

Information about you and other information obtained during the study will be kept confidential. However, information containing your personal data may be disclosed in certain circumstances. For example, at any time after the end of the study, representatives of medical authorities or members of the ethical committee that approved the study may review the research documentation or medical records to ensure that the results obtained during the study are accurately documented.

10. Is anyone monitoring the research?

There are International documents that regulate the conduct of clinical trials to ensure their safety. They are called "Good Clinical Practice" and "Declaration of Helsinki". This study is conducted according to these standards.

11. What happens after the end?

After your participation in the study, you will be given standard treatment depending on the histological nature of the tumor and in accordance with the standards for providing care to patients with brain tumors approved by the Russian Ministry of Health.

12. Contacts for additional information.

Thank you for reading this information. If you do not understand anything or have additional questions, please contact your doctor as soon as possible.

Contact information:

Signature ___________

Other employees of the institute:

Medyanik Igor Aleksandrovich
Address Nizhny Novgorod, Verkhne-Pecherskaya embankment 18
Phone +7 9087676221

Patient's signature ___________ Date _________________
